# Iron-modified biochar derived from sugarcane bagasse for adequate removal of aqueous imidacloprid: sorption mechanism study

**DOI:** 10.1007/s11356-022-22357-6

**Published:** 2022-08-16

**Authors:** Yongliang Chen, Masud Hassan, Md Nuruzzaman, Huiming Zhang, Ravi Naidu, Yanju Liu, Ling Wang

**Affiliations:** 1 School of Resources and Environmental Engineering, Wuhan University of Science and Technology, Wuhan, China; 2 Global Centre for Environmental Remediation, College of Engineering, Science and Environment, The University of Newcastle, Callaghan, NSW 2308 Australia; 3 CRC for Contamination Assessment and Remediation of the Environment (CRC CARE), Callaghan, NSW 2308 Australia; 4 Cooperative Research Centre for High Performance Soil (CRC SOIL), IDB Building, The University of Newcastle, Callaghan, NSW 2308 Australia; 5 Electron Microscope and X-Ray (EMX) Unit, The University of Newcastle, Callaghan, NSW 2308 Australia

**Keywords:** Adsorption, Biochar, Pesticides, Removal, Waste materials, Wastewater

## Abstract

Adsorption has been considered as a promising remediation technology to separate organic and inorganic agrochemicals from contaminated soil and water. Low-cost adsorbents, including waste derived materials, clay composites, biochar, and biochar modified materials, have attracted enormous attention for the removal of organic contaminants, including pesticides. In this study, iron-modified base-activated biochar (FeBBC) was prepared by pyrolysis (at 400 °C for 1 h) of iron-doped base (KOH) activated sugarcane bagasse for the removal of a widely used insecticide, namely imidacloprid (IMI) from water. The maximum adsorption capacity of the adsorbent (FeBBC) was calculated as 10.33 (± 1.57) mg/g from Langmuir isotherm model. The adsorbents could remove up to ~ 92% of IMI from aqueous solution at 23.8 mg/L IMI. Experimental data fitted well with the Freundlich model and pseudo-second-order model, demonstrating physisorption, as well as chemosorption, contributed to the sorption process. Even at highly acidic/basic solution pH, the FeBBC could remove substantial amount of IMI demonstrating hydrophobic interaction and pore diffusion play vital role for removal of IMI. The slight improving of IMI sorption with increasing solution pH indicated the sorption was also facilitated through ionic interaction alongside physical sorption. However, physical sorption including hydrophobic interaction and pore-filling interaction plays a vital role in the sorption of IMI.

**Supplementary Information:**

The online version contains supplementary material available at 10.1007/s11356-022-22357-6.

## Introduction

In agriculture, pesticides play an important role in crop production by protecting plants and crops from weeds, insects, and diseases (Cooper and Dobson 2007; Saravi and Shokrzadeh 2011). However, it is important to ensure their safe application because of continuous exposure causes serious problems to non-target organisms (Nuruzzaman et al. 2016). For instance, the first neonicotinoid insecticide, imidacloprid (1-(6-chloro-3-pyridylmethyl)-*N*-nitro-imidazolidin-2-ylideneamine), was introduced in 1991 (Tomlin 2009) and has become one of the most successful insecticides that occupies a share of about 25% of the overall insecticide market (Bass et al. 2015; Ma et al. 2021d). So far, its application has been observed in more than 120 countries and over 140 crops, including maize, cotton, wheat, potatoes, and rice (Bass et al. 2015; Jeschke and Nauen 2008; Nauen et al. 2008; Tomizawa and Casida 2005). Because of its huge application, the global contamination of surface water with imidacloprid (IMI) has been observed over the last decade (Johnson and Pettis 2014; Morrissey et al. 2015; Van Dijk et al. 2013; Zhang et al. 2019). Recent studies also exhibited the toxic effects of IMI and its metabolites on various aquatic organisms (Sumon et al. 2018; Van Dijk et al. 2013). Thus, IMI is considered a potential organic contaminant and listed as 2nd of European priority substances (Barbosa et al. 2016; Loos et al. 2018; Ma et al. 2021d; Regulation 2009). The concentration of IMI in the natural system often exceeds the regulatory guidelines of the USEPA’s aquatic life benchmark value of 1.05 µg L^−1^ (Morrissey et al. 2015; Starner and Goh 2012). Therefore, it is critical to remove IMI from water bodies that have created significant attention to the research community.

To this aspect, a set of technologies are used to eliminate IMI from water, including advanced oxidation, photo-catalysis, biodegradation, and adsorption (Hayat et al. 2020; Kalhor et al. 2018; Phugare et al. 2013; Rodriguez-Narvaez et al. 2017; Wang et al. 2020). Of these technologies, adsorption has been considered a promising technology for the removal of pesticides due to low operation cost, reusability, simplicity, removal efficiency, reluctant to induce secondary daughter contaminations, and modest environmental impact. To date, various adsorbents, including graphene, clay, biochar, porous silica, and polymer composites, were used to adsorb IMI from aqueous solution (Ishtiaq et al. 2020; Kalhor et al. 2018; Keshvardoostchokami et al. 2018; Ma et al. 2021c; Ma et al. 2021d; Mandal et al. 2017; Mohammad and El-Sayed 2020; Nuruzzaman et al. 2019; Singh et al. 2021). Among these adsorbents, biochar (BC) has held a prominent position in agricultural practices because of its multipurpose application including mulching (Beesley et al. 2011) and carrier materials for agrochemicals (Sashidhar et al. 2020). It has been observed that the application of biochar to soil prevents leaching of pesticide residues (Giori et al. 2014; Li et al. 2014). Additionally, it can be synthesized easily through the process of carbonification of biomass (Jeon et al. 2021). However, several factors affect the performance and physicochemical properties of BC including feedstock sources as well as pyrolysis temperature. For instance, grass-derived biochar requires lower pyrolysis temperature to produce hydrophobicity than hard wood-derived biochar which is crucial to adsorb organic molecules (Hassan et al. 2020b).

Preparation of BC from agricultural wastes potentially could be a sustainable and environmentally friendly option. For example, sugarcane bagasse (SB) could be used as feedstock source material for large-scale biochar production due to its global abundance, low cost, high mineral content, and porous fibre structure (Hassan et al. 2022a, 2021). In addition, it contains a higher content of cellulose and hemicellulose compared to lignin which facilitates the abundance of functional groups onto biochar. Generally, a lower percentage of lignin makes this feedstock (SB) lower thermal stability resulting low yield of biochar. The adsorption capacity of BC could be increased by their surface activation or modification. Ma et al. reported that base-activated BC showed higher removal efficiency for IMI than raw BC (Ma et al. 2021b). Moreover, iron-modified BC has a higher sorption capacity than unmodified biochar for the removal of IMI (Ma et al. 2021c, 2021d). The presence of iron during BC preparation facilitates their physicochemical properties. Such as degradation of the aliphatic carbon phase occurs faster in the presence of iron (Liu et al. 2022; Nguyen et al. 2021). As a result, rapid carbonization of biomass generates hydrophobicity onto carbonaceous materials, consequently, sorption performance is enhanced via hydrophobic interaction for organic molecules. Iron-modified adsorbents could also enhance ionic interaction with charged organic and inorganic contaminants (Hassan et al. 2022a, 2022b).

Hence, the key objective of this study is to synthesize iron-doped base-activated biochar (FeBBC) using SB as a promising feedstock and investigate the sorption of IMI. A comparison of physicochemical properties of base-activated biochar (BBC) and FeBBC was also presented to understand the role of Fe-doping onto the properties of metal-modified biochar. Furthermore, effects of solution pH, active sites, the concentration of solutes (isotherm), and contact time (kinetics) for IMI adsorption onto the FeBBC were investigated to understand the adsorption mechanism and removal efficiency for IMI. This study provides a fundamental understanding of IMI sorption onto Fe-doped carbonaceous material, which could be utilized to promote future research on pesticide removal.

## Materials and methods

### Chemicals and feedstock

All the required chemicals, i.e. ferrous chloride, ferric chloride, potassium hydroxide (KOH), sodium hydroxide (NaOH), hydrochloric acid (HCl), and imidacloprid (PESTANAL, 99.99% purity), were obtained from Sigma-Aldrich, Australia. Milli-Q water (18.2 MΩ) was used in all experiments. The raw sugarcane bagasse (SB) was obtained from Sunshine sugar mill, NSW, Australia. The raw SB was milled into particles followed by sieving using a stainless-steel sieve (sieve opening 4 mm). The sieved SB was dried up under sunlight in a glasshouse for 14 days before any modification. The physicochemical properties, molecular structure, and ionization behaviour of IMI are presented in the supplementary information (See Table SM1, Scheme SM1, and Fig. SM1).

### Synthesis of adsorbent

**Scheme 1:**
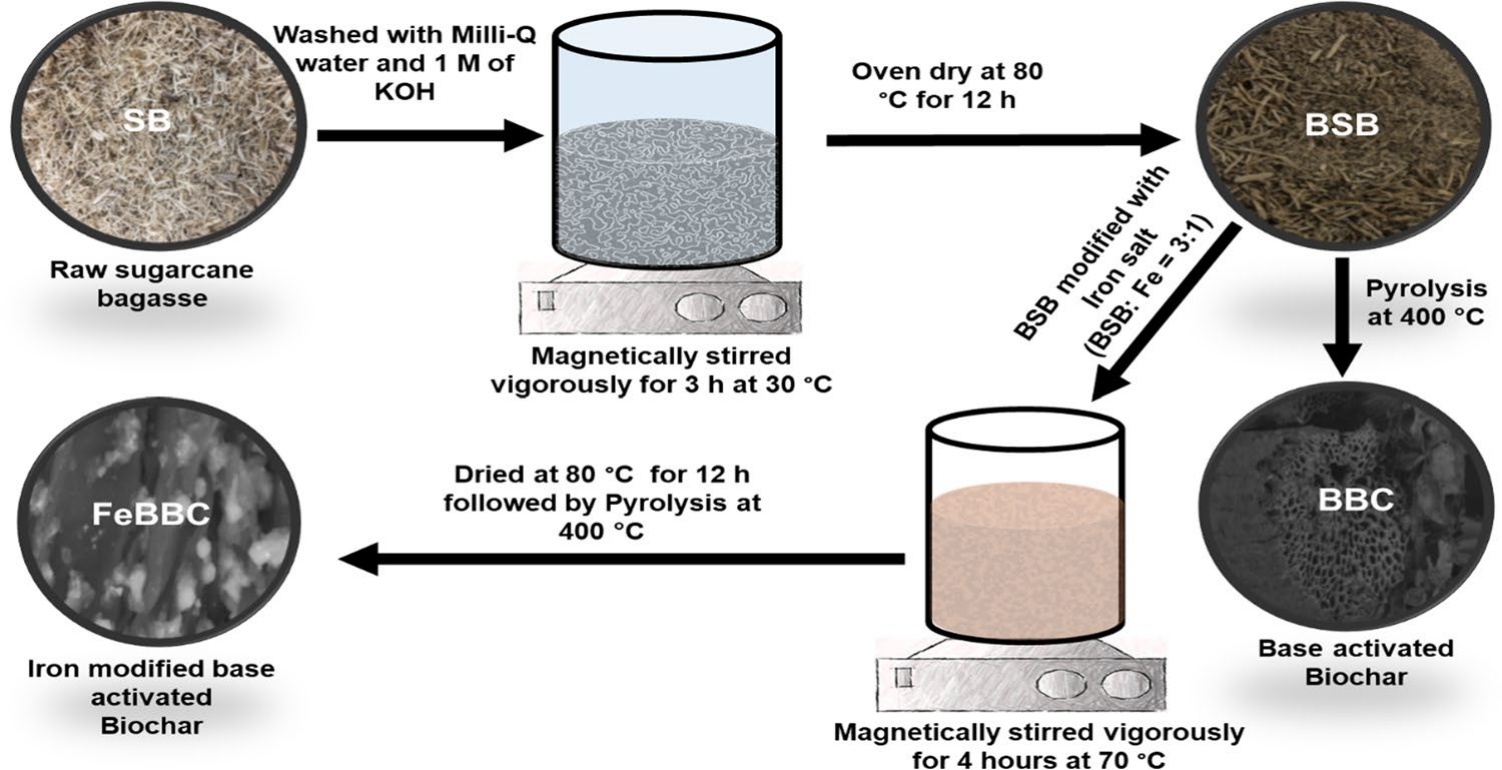
Synthesis procedure of base-activated biochar (BBC) and base-activated iron-modified biochar (FeBBC)

A simple single-step method was utilized to prepare Fe-modified base-activated biochar (FeBBC). The synthesis procedure has been presented in Scheme 1. In brief, 100 g of dried SB (particle size < 4 mm) was washed using Milli-Q water to eliminate dust and other wreckage. The washed SBs were immersed into 500 mL of 1.0 M KOH solution in a 1 L glass beaker and homogenized at 120 rpm for 3 h using a hot plate magnetic stirrer at 30 °C. The base-activated SB (BSB) was then washed with Milli-Q water until the pH of reinstate reached close to ~ 7, followed by oven drying at 80 °C for 12 h. The oven-dried BSB was then crushed using a ceramic mortar pestle and sieved (< 1 mm) before modification with ferrous and ferric chloride (at the ratio of Fe^2+/^Fe^3+^  = 5:1) solution.

To induce Fe particles onto BSB, 100 g of dried BSB was immersed into 300 mL of Fe^2+/^Fe^3+^ solution where the mass ratio of BSB and Fe was 3:1. To achieve a homogeneous mixture, the system was stirred vigorously using a hot plate magnetic stirrer under oxygen-limited conditions for 4 h at 70 °C. The iron-modified BSB was separated from the mixer by filtration and washed with Milli-Q water several times. Consequently, the iron-modified BSB was dried out in an oven at 80 °C for 12 h. The dried samples were stored in an airtight condition before pyrolysis. The pyrolysis of iron-treated BSB and untreated BSB was conducted at 400 °C (for 1 h) in a muffle furnace with an incremental heating rate of 10 °C min^−1^ under nitrogen gas conditions. The pyrolysed samples were collected after cooling overnight to below 50 °C. The collected based activated biochar and iron-modified base-activated biochar are denoted as BBC and FeBBC respectively. The characterization method of the adsorbents has been highlighted in Supplementary text I.

### Batch adsorption studies and data analysis

To understand the adsorption process of IMI onto FeBBC, isotherm, kinetics, influences of pH, and adsorption dose studies have been conducted using batch studies. Initially, stock solution of IMI at 100 mg/L was prepared which was used for adsorption studies with proper dilution. The sorption studies were performed at 150 rpm in an end-over-end shaker at 25 (± 1) °C. The effect of adsorbent dose was conducted from 0.5 to 10 g/L of adsorbents, where C_0_ was 23.0 (± 0.1) mg/L and contact time was 21 h. Whereas the batch isotherm study was carried out using IMI concentration ranging from 1 to 100 mg/L where the contact time (t) was 21.0 h, the adsorbent dose was 1.67 g/L, and the system pH was adjusted as 8.0. The kinetics study was conducted up to 72 h where initial pesticide concentration (C_0_) was 23.8 mg/L, initial solution pH was 3.0, and adsorbent dose was 2.5 g/L. The influences of pH on IMI sorption were conducted at solution pH from 2 to 10, where, contact time (t) was 21 h, C_0_ was 23.8 mg/L, and adsorbents dose was 2.5 g/L. The solution or system pH was altered by using 0.1/0.01/0.001 M NaOH or HCl solution.

After the sorption processes, the samples were centrifuged at 3000 rpm for 15 min to separate the adsorbents for concentration analysis. The supernatant of each sample was further filtered (0.22 μm, cellulose acetate membrane), then the residual concentrations of IMI in solution were determined using a UV–Vis spectrophotometer (Shimadzu UV3600 Plus) at λ_max_ of 270 nm. A standard calibration curve (*R*^2^ = 0.997) was developed for IMI measurement and calculations (see Fig. SM2 in the supplementary information). All the experiments were conducted in duplicates, and mean values were presented with a standard deviation. The removal percentage (*R*, %) and amount of IMI adsorbed at time *t* (*Q*_*t*_) and equilibrium (*Q*_*e*_) were calculated using the following equations, respectively:

1 $$\% R= \frac{({C}_{0 }-{C}_{e})}{{C}_{0}}\mathrm{x }100$$

2 $${Q}_{t}= \frac{({C}_{0 }-{C}_{t})V}{m}$$

3 $${Q}_{e}= \frac{\left({C}_{0 }-{C}_{e}\right)V}{m}$$

where *C*_*0*_, *C*_*t*_, and *C*_*e*_ are the *initial*, *t* time, and *equilibrium* solution concentrations (mg L^−1^), respectively; *Q*_*t*_ and *Q*_*e*_ are the amounts of pesticide adsorbed per gram of adsorbent (mg g^−1^) at time *t* and equilibrium, respectively; *V* is the volume of solution (L), and *m* is the mass of the adsorbent (g).

## Result and discussion

### Physiochemical properties of FeBBC

Physiochemical properties and yield of biochar depend on the feedstock sources (e.g. hardwood, softwood, manure, grass) and pyrolysis temperature (Hassan et al. 2020b; Mukome et al. 2013). Usually, biochar yield, hydrophilicity, carbon, and oxygen content are reduced with increasing pyrolysis temperature whereas inorganic (silicon, sulphur, potassium) percentage depends on raw biomass sources (Hassan et al. 2020b). Furthermore, the structural composition (cellulose, hemicellulose, and lignin) of plant biomass is the prime indicator for thermal stability, where the higher lignin content is responsible for a higher yield of biochar. Generally, SB contains about 40–50%, 25–35%, and 15–35% of cellulose (glucose polymer cellulose), hemicellulose (amorphous polymer consisting of xylose, arabinose, galactose, glucose, and mannose), and lignin (crystalline carbon) respectively. It also contains a lower amount of minerals, wax, and other compounds (less than 1% on a dry weight basis) (Hassan et al. 2020b; Starner and Goh 2012). Thus, the yield of sugarcane bagasse-derived biochar (grass-derived biochar) is comparatively lower than hardwood and softwood-derived biochar due to low thermal stability resulting from the lower lignin content. A lower yield percentage (22.81%) was observed in base-activated biochar (BBC), whereas the yield percentage was increased to 43.42% for iron modified biochar (FeBBC) (Table 1).

**Table 1:** Properties of the base-activated biochars before (BBC) and after iron modification (FeBBC)

Properties	Biochars	
	BBC (base activated biochar)	FeBBC (iron modified base activated biochar)
Pyrolysis conditions	HHT: 400 °C, pyrolysis time: 1 h, N_2_ condition	HHT: 400 °C, pyrolysis time:1 h, N_2_ condition
Yield (%)	22.81	43.42
Mass loss (%)	77.18	56.57%
C content (wt. %)	58.47	40.27
S content (wt. %)	0.34	0.05
N content (wt. %)	0.34	0.29
Minerals phase	Quartz, amorphous carbon	Quartz, amorphous carbon, iron

However, an opposite trend was observed in elemental content due to introduction of iron. It was observed that FeBBC contains lower carbon (C), sulphur (S), and nitrogen (N) content compared to BBC (Table 1). The presence of iron during pyrolysis process also enhances the catalytic degradation of the aliphatic carbon phase resulting in lower carbon content but higher hydrophobicity. It also facilitates rapid volatilization of amorphous carbon phase resulting reduction of weight percentage (wt.%). It was also reported that higher cellulose and hemicellulose content promotes higher oxygen content containing functional groups in biochar which depends on the pyrolysis intensity and condition. Therefore, it is expected that biochar derived from SB (BBC and FeBBC) will be enriched with oxygen-containing active sites because it contains higher content of cellulose and hemicellulose than lignin (Hassan et al. 2020b; Starner and Goh 2012).

**Fig. 1:**
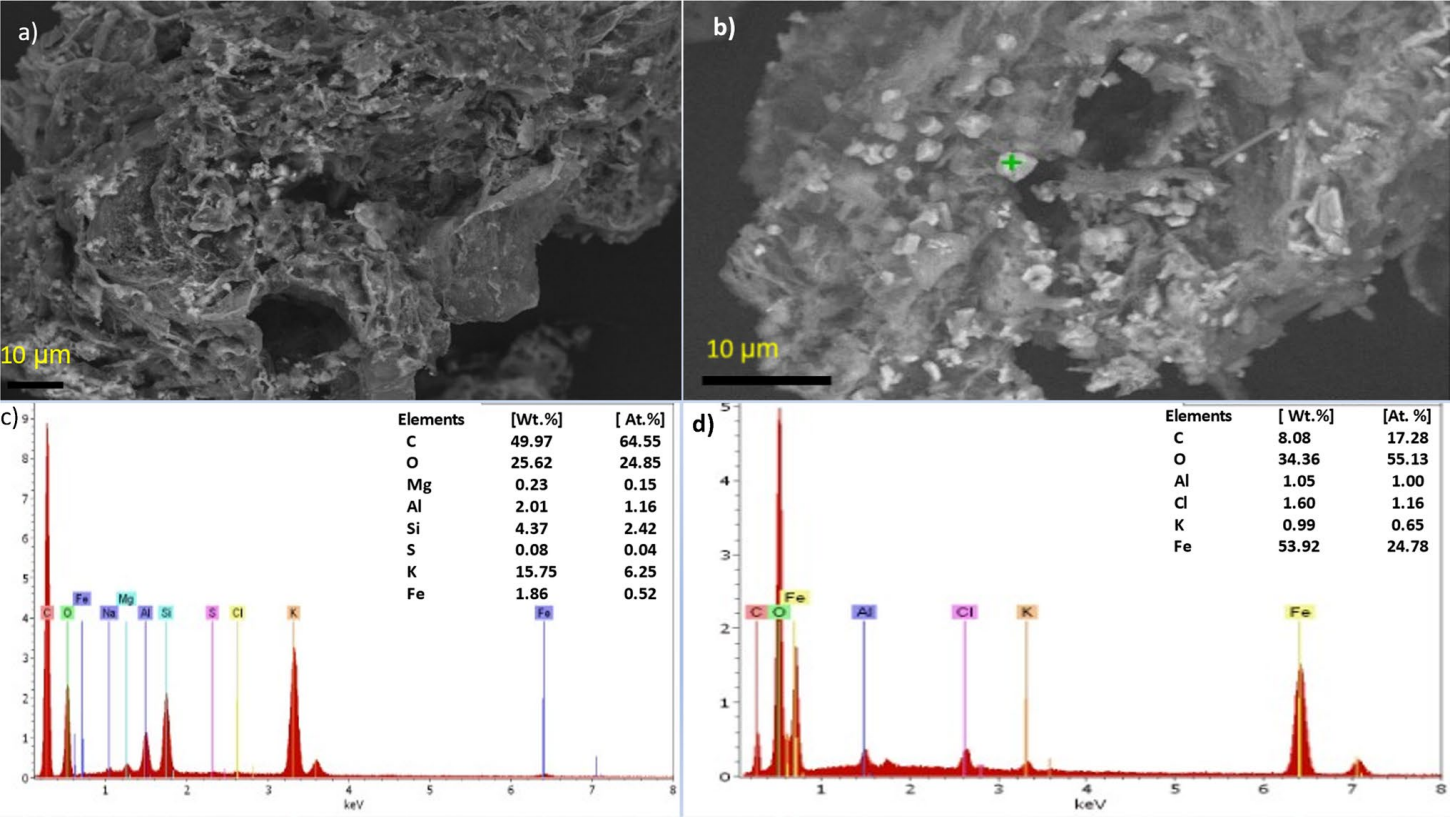
Scanning electron microscope (SEM) images of BBC (**a**) and FeBBC (**b**); as well as EDS elemental spectra with elemental compositions (as weight (Wt.) % and atomic (At.) % of BBC (**c**) and FeBBC (**d**)

Furthermore, SEM images revealed the porous structures in both BSB and FeBBC (Fig. 1a and b) where the Fe particles were located and stabilized in and around the porous structure in FeBBC (Fig. 1b). The presence of iron in FeBBC was also confirmed by EDS analysis. The EDS spectra of BBC and FeBBC and their respected elemental compositions are presented in Fig. 1c and d respectively. A tiny amount of Fe was identified in BBC whereas the Fe content profoundly increased in FeBBC. Alongside the aliphatic carbon phase, a trace amount of silicon, potassium, and aluminium was also found in BBC and FeBBC (Fig. 1c and d). The mineral phases could provide active functional groups for adsorption of organic and inorganic molecules.

**Fig. 2:**
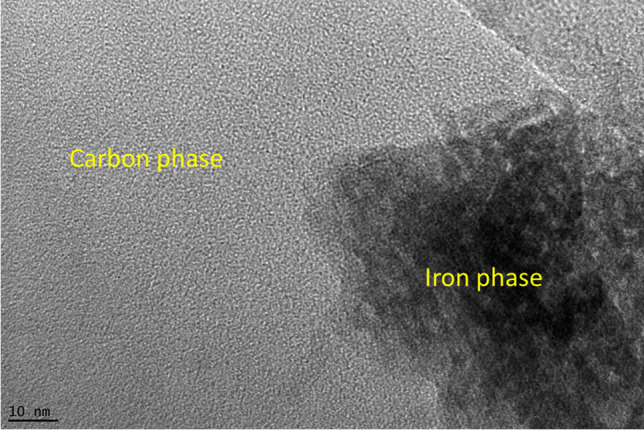
Transmission electron microscope (TEM) image of iron-modified biochar (FeBBC)

The presence of Fe in FeBBC was identified from the XRD pattern indicated the doping of Fe onto base-activated SB-derived biochar (see Fig. SM3 in the supplementary information). In addition, quartz (SiO_2_) was identified in the XRD pattern (see Fig. SM3 and Table SM2 in the supplementary information). The amorphous carbon phase of cellulose and hemicellulose in SB was recognized at 2Ɵ = 16.68°. TEM images are also clearly show the presence of amorphous carbon structure in FeBBC (Fig. 2). With increasing the highest heating temperature (HTT), the cellulose and hemicellulose started to decompose and volatilize. The relative oxygen content is reduced, whereas relative carbon increased due to thermal treatment, which is justified by the EDS spectra of BBC and FeBBC (see Fig. 1). Carbon skeletal appeared to form due to the rapid decomposition of cellulose and hemicellulose. More order aromatic carbon skeleton appeared over 600 °C for hardwood (Hassan et al. 2020a), whereas for SB-derived biochar appears as aromatic carbon structure from amorphous carbon phase at relatively lower HTT (Fig. 2).

### Removal of IMI by FeBBC

#### Effect of adsorbent dose

**Fig. 3:**
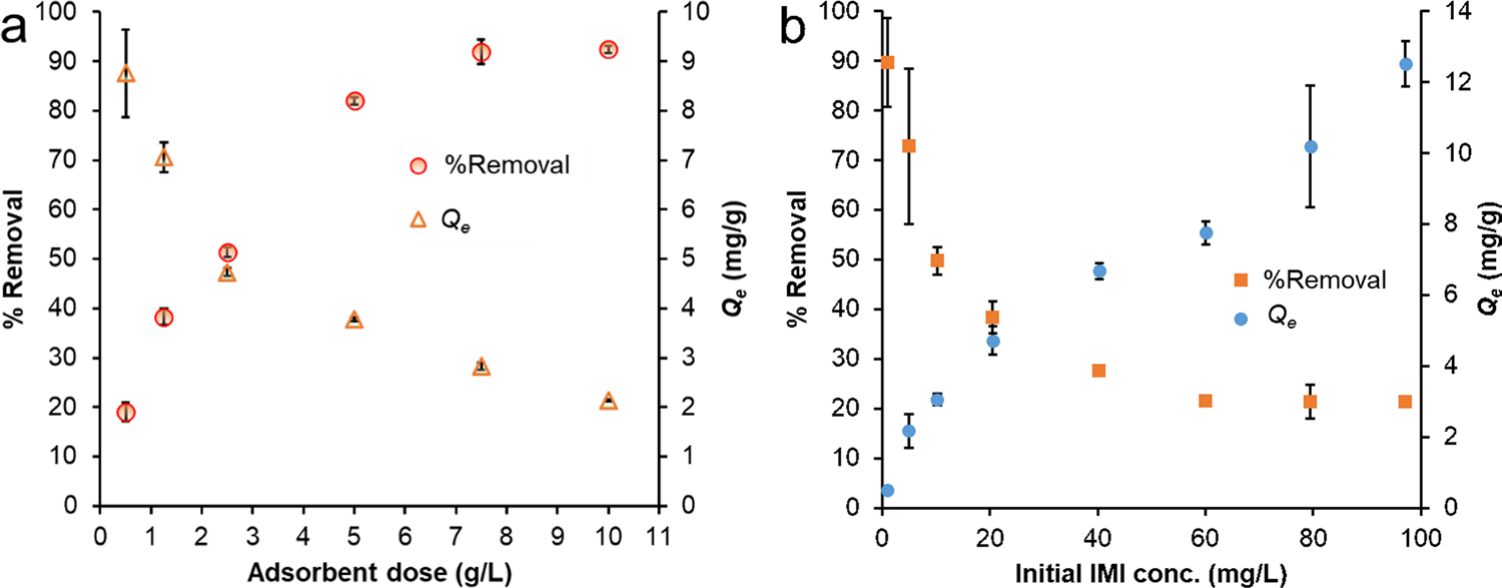
Effect of (**a**) the adsorbents dosage and (**b**) initial IMI concentration on IMI removal by FeBBC

The dose study of the adsorbents was performed to determine the influence of FeBBC amount on removal % of IMI from aqueous solution. The removal percentage of IMI increased considerably from ~19 to 92% with the rise in adsorbent dosage from 0.5 to 7.50  g/L (Fig. 3a). The acceleration in IMI removal with increasing dosage of adsorbent was due to the availability of active sites. However, the amount of adsorbed IMI decreased considerably, while the adsorbent dosage raised from 0.5 to 10 g/L. The adsorption capacity of IMI reduced with increasing adsorbent dose due to a relative decrease in the amount of IMI adsorbed per unit of adsorbent resulting from higher adsorbent doses. Over the saturation of the adsorbent’s active sites, the sorption of IMI did not increase significantly. Therefore, the adsorbents dose of 7.5 g/L can effectively remove more than 90% IMI along with a concentration of 23.0 (± 0.1) mg/L from water.

#### Effect of initial IMI concentration

The effect of initial IMI concentration on IMI removal and adsorption is shown in Fig. 3b. Batch sorption results suggested that the removal efficiency of FeBBC varied from ~90 to 21% for IMI with initial concentrations ranging from 1.0 to 100 mg/L. However, the amount of IMI adsorbed by FeBBC increased with increasing initial concentrations of IMI. The decrease in removal with a high concentration of IMI may attribute to a less amount of available active sites. The amount of IMI adsorbed increases almost linearly with the increase of IMI concentration. The increasing trend of IMI concentration afforded to mass transfer of IMI. In this case, the portioning behaviour of IMI played an important role.

#### Effect of solution pH

**Fig. 4:**
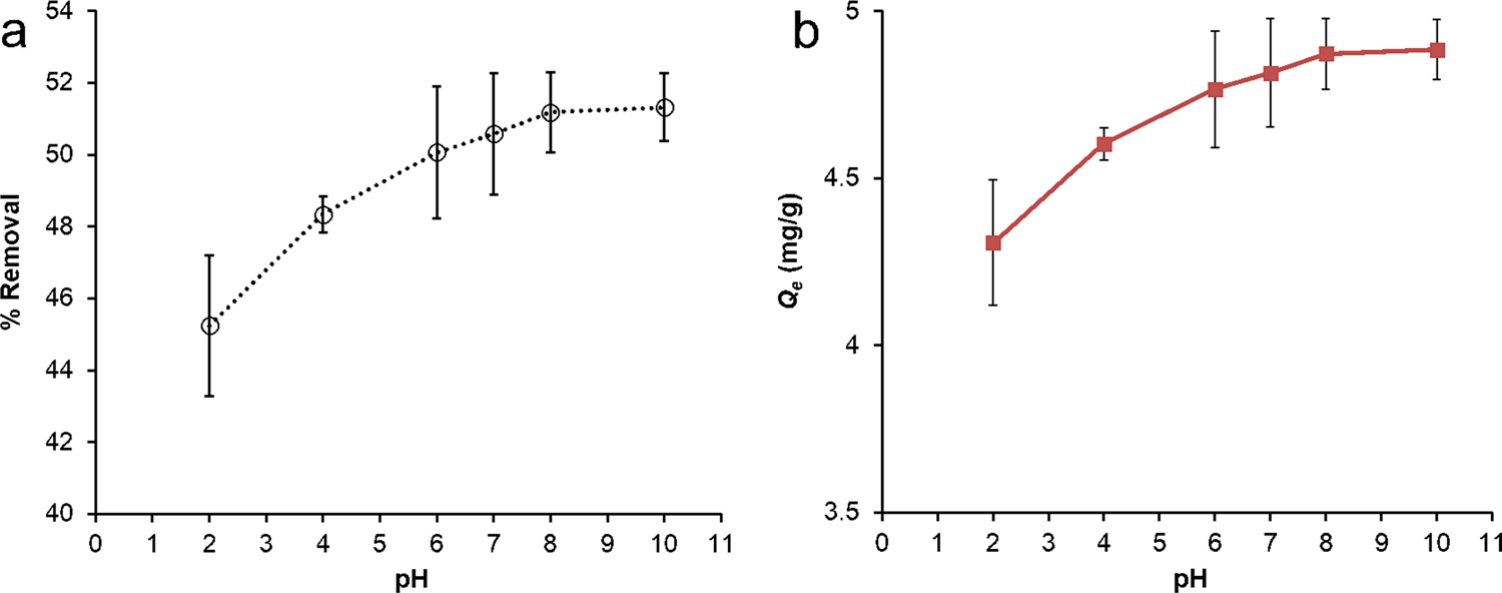
Effect of solution pH on IMI removal by FeBBC from water

Solution pH is an important factor for the surface charge properties of the adsorbents and ionization of adsorbates. A competitive adsorbent should have optimum sorption capacity at a wide pH range. Thus, the influence of the initial solution was investigated in the range of pH 2–10 using 23.8 mg/L of IMI. It was observed that the IMI removal efficiency was in the range 45–51% which represented the adsorbed amount 4.3 to 4.9 mg/g over solution pH range of 2–10 (Fig. 4). Dunnett analysis indicated no significant changes in adsorption amount over the pH range investigated. Therefore, the effect of solution pH at 2–10 on IMI adsorption was insignificant, and the FeBBC could effectively adsorb IMI from water at a wide range of pH.

### Sorption isotherm

Langmuir and Freundlich isotherm models were used for evaluating the properties of the deposited layer of IMI over the active sites of adsorbents (Freundlich 1907; Langmuir 1918; Worch 2012). The non-linear form of Langmuir and Freundlich isotherm model is given in Supplementary text II.

**Fig. 5:**
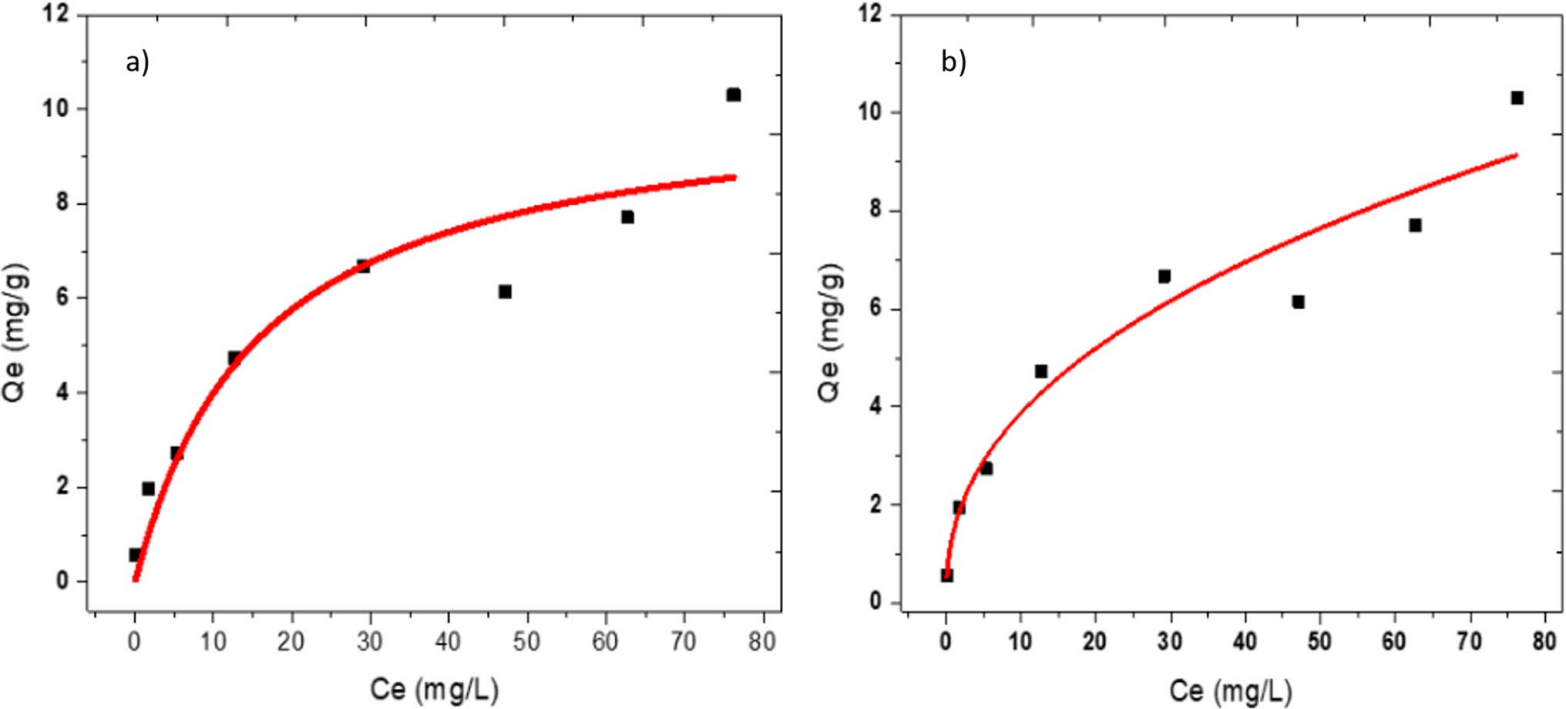
Isotherm model fitting of IMI sorption onto iron-modified biochar; **a**) Langmuir isotherm model fitting. **b**) Freundlich isotherm model fitting

The experimental data fitted well to the Freundlich isotherm model (*R*^2^ = 0.93) compared to the Langmuir isotherm model (*R*^2^ = 0.89) (Fig. 5). The isotherm model fitting demonstrated that IMI sorption occurs through heterogeneous multilayer sorption over the adsorbents’ active sites. The adsorption capacity of IMI increased fast initially due to the abundance of active sites that demonstrated higher selectivity of IMI onto the adsorbents. The increasing trend of adsorption capacity of IMI decreased over equilibrium concentration about 30 mg L^−1^. The maximum sorption capacity of IMI to FeBBC was determined at about 10.34 mg g^−1^ from the Langmuir isotherm model. The *K*_L_ values lie between 0 and 1 which demonstrated the sorption of IMI is spontaneous and favourable, which is suitable for sorption. As the value of 1/*n* reaches close to 1, the isotherm becomes linear due to homogeneous surface (Haghseresht and Lu 1998; Tan et al. 2008). The microscopic images (SEM and TEM) of the adsorbent indicated that the adsorbent’s surfaces are heterogeneous due to the incorporation of iron over the heterogeneous surface of the biochar (Haghseresht and Lu 1998; Tan et al. 2008).

### Kinetic evaluation of IMI sorption to FeBBC

**Fig. 6:**
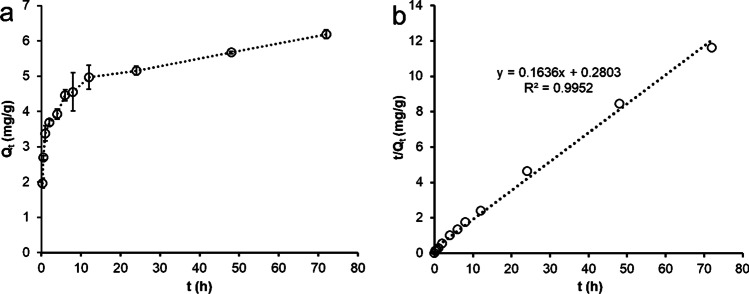
IMI sorption kinetics; **a** effect of contact time on adsorption of IMI onto FeBBC; **b** pseudo second order model fitting (linear fitting)

Time-dependent sorption of IMI onto FeBBC was evaluated for 72 h of contact time (Figs. 6a and SM4). It was observed that IMI sorption occurred in mainly three identical stages. Initially, rapid sorption occurs, where ~55% of the total adsorbed amount is adsorbed by 1 h. After initial rapid adsorption due to physical sorption of IMI, a comparatively slower adsorption kinetics observed up to 12 h of contact time. Remaining IMI adsorbed at extremely slow rate due to slower diffusion kinetics of IMI molecules onto the porous interior of the FeBBC (see Fig. 6a). As the sorption kinetics is not linear with contact time and has three identical stages, demonstrated multiple sorption mechanisms were taken place during the sorption process of IMI. Furthermore, to understand the overall sorption process, experimental data were further analysed using pseudo-first-order (PFO) and pseudo-second-order (PSO) kinetic models, and intra-particle diffusion models were highlighted in supplementary text II.

The pseudo-second kinetic model fitted well (*R*^2^ = 0.99) than the pseudo-first-order kinetic model demonstrating both physisorption and chemosorption of IMI. The pseudo-first-order kinetic model explained either physisorption or chemisorption could take place during the adsorption process. In contrast, the pseudo-second-order kinetics model showed both physisorption and chemisorption of the adsorbate, and the amount of IMI adsorbed onto the composite at equilibrium condition (Q_e_) is 6.19 mg/g (Table 2), which is close to the actual experimental value (Fig. 6a).

**Table 2:** Isotherm and kinetics parameters of IMI sorption on FeBBC

Isotherm and kinetics models	Parameters	FeBBC^*^
Langmuir isotherm model	Q_m_ (mg/g)	10.34 (± 1.51)
	K_L_ (L/mg)	0.06 (± 0.03)
	*R*^2^	0.89
	Chi-square	1.18
Freundlich isotherm model	K_F_ (mg/g)/mg/L)^n^	1.47 (± 0.38)
	1/n	0.42 (± 0.07)
	*R*^2^	0.93
	Chi-square	0.70
Pseudo-first-order kinetic model	Q_e_ (mg/g)	4.07
	K_1_ (h^−1^)	0.20
	*R*^2^	0.84
Pseudo-second-order kinetic model	Q_e_ (mg/g)	6.19
	K_2_ (g/mg. h)	0.38
	*R*^2^	0.99
Intra-particle diffusion model	Q_e (_mg/g)	0.62
	K_α_ (mg /g. min^1/2^)	0.36
	*R*^2^	0.99
	Q_e_ (mg/g)	2.74
	K_β_ (mg /g. min^1/2^)	0.08
	*R*^2^	0.98
	Q_e_ (mg/g)	4.04
	K_γ_ (mg /g. min^1/2^)	0.03
	*R*^2^	0.97

^*^Values within bracket indicates standard deviation

**Fig. 7:**
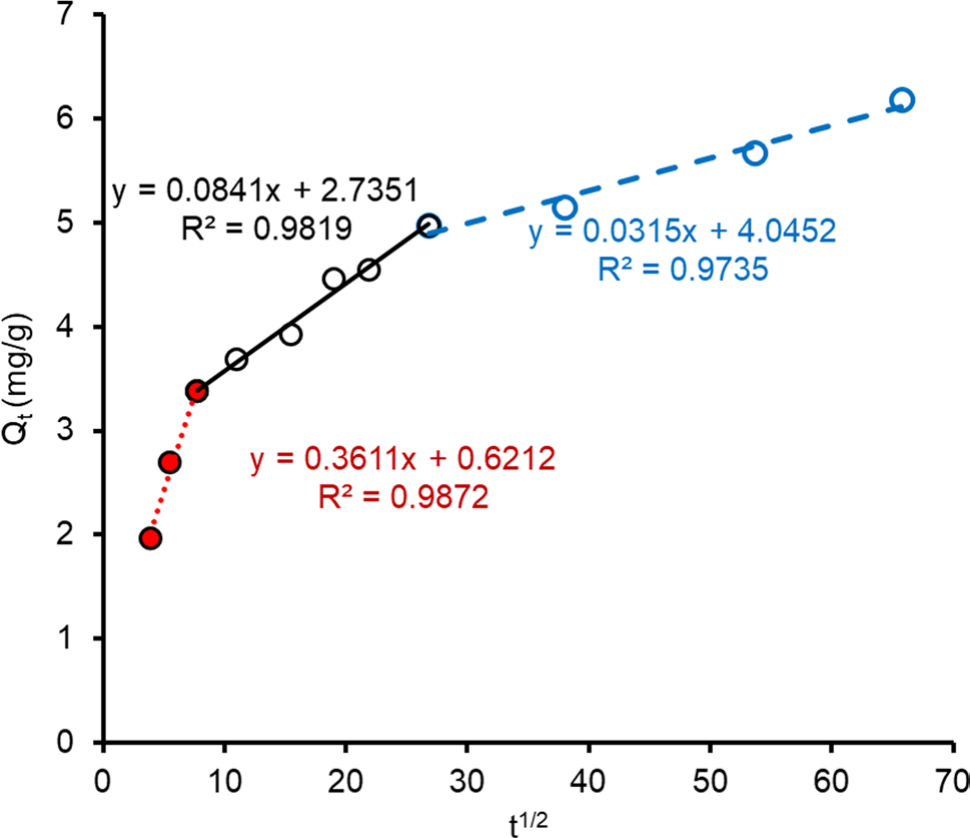
Intra-particle diffusion plot for imidacloprid (IMI) adsorption onto the adsorbent (FeBBC)

The intra-particle diffusion rate constant (*k*_*p*_) found from the slope of linear gradients of the plots of *Q*_*t*_ versus *t*^½^ (Fig. 7) was presented in Table 2. Figure 7 demonstrated the adsorption proceeds via a more complex mechanism including surface adsorption and pore diffusion (Wu et al. 2005). The intercept observed in the linear segment indicates that pore diffusion was not the controlling step for IMI sorption. Boundary layer effect—might diffuse IMI molecules. Thus, the kinetics of IMI adsorption to FeBBC reflect a highly hydrophobic interaction and low diffusion rates. The adsorption process fails to attain an equilibrium state when both chemical non-equilibrium and rate-limiting diffusive mechanisms are engaged in sorption (Brusseau et al. 1991). The rate-limiting diffusive mass transfer could be involved intra-particle as well as intra-sorbent diffusion. The first phase could be described as rapid adsorption of imidacloprid on the external surface of FeBBC and/or phase partitioning properties of pesticide. The second phase and third phase could be described as the gradual adsorption stages, where adsorption of imidacloprid occurred via intra-particle diffusion (Wu et al. 2005).

### IMI sorption mechanism

**Fig. 8:**
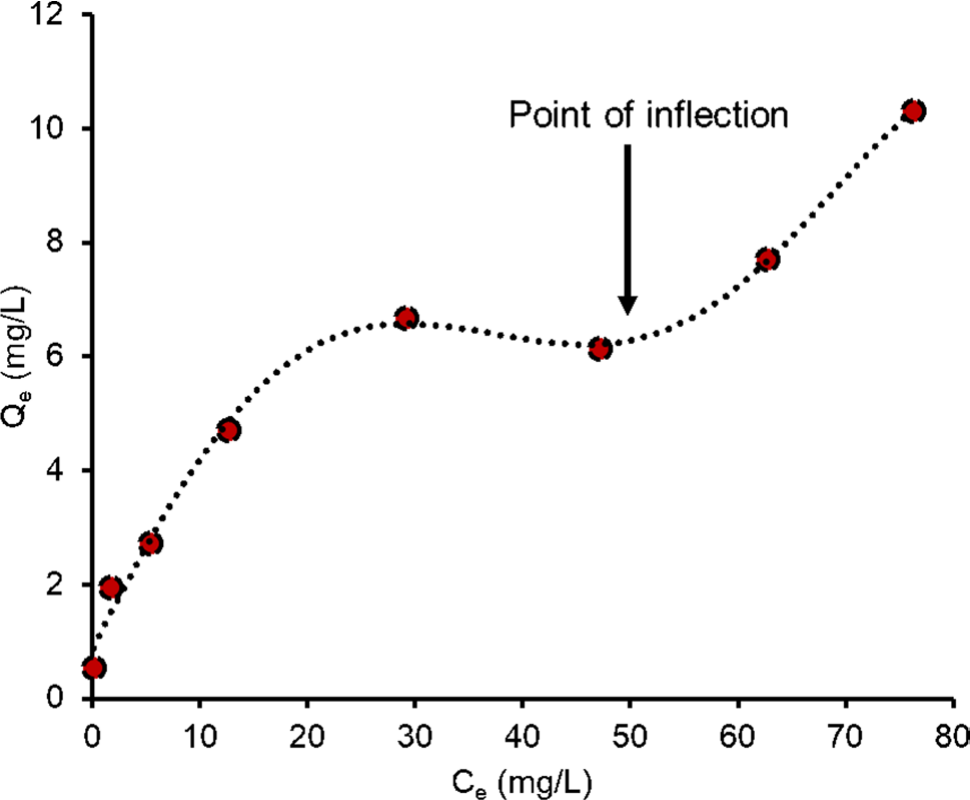
Sigmoidal isotherm curve of IMI sorption onto FeBBC

The sorption isotherm indicated that FeBBC processes a heterogeneous surface where the sorption of IMI onto FeBBC might take place via a number of mechanisms. Basically, the sorption isotherm prevailed sigmoidal curve and indicates a point of inflection (Fig. 8). Such isotherm also results from at least two opposite mechanisms where the point of inflection explains the concentration required to overcome the complexation during sorption process (Limousin et al. 2007). This may be due to partitioning behaviour of IMI where initially a low amount adsorbed to FeBBC surface and further, adsorption enhanced by interaction among IMI. The other possible reason is the presence of both polar (metal ligands) and non-polar (amorphous C) moieties in FeBBC.

Whereas the sorption kinetics showed three certain stages during IMI sorption into FeBBC, initially, rapid sorption occurred due to physical sorption including hydrophobic interaction as well as π-π conjugation among the FeBBC and IMI molecules, high IMI concentration, and availability of abundance of unoccupied active sites. After that, polar interaction may take place to adsorb IMI from the aqueous phase, which is relatively slower than hydrophobic and π-π interaction, but faster than intraparticle diffusion. The last stage of IMI sorption was governed by intraparticle diffusion. The size of IMI is 10 × 6 × 5 Å, which enables intra-particle diffusion could play a vital role in the adsorption of IMI from water (De Smedt et al. 2015). Intra-particle diffusion model indicated the presence of both macro-and micropores.

**Fig. 9:**
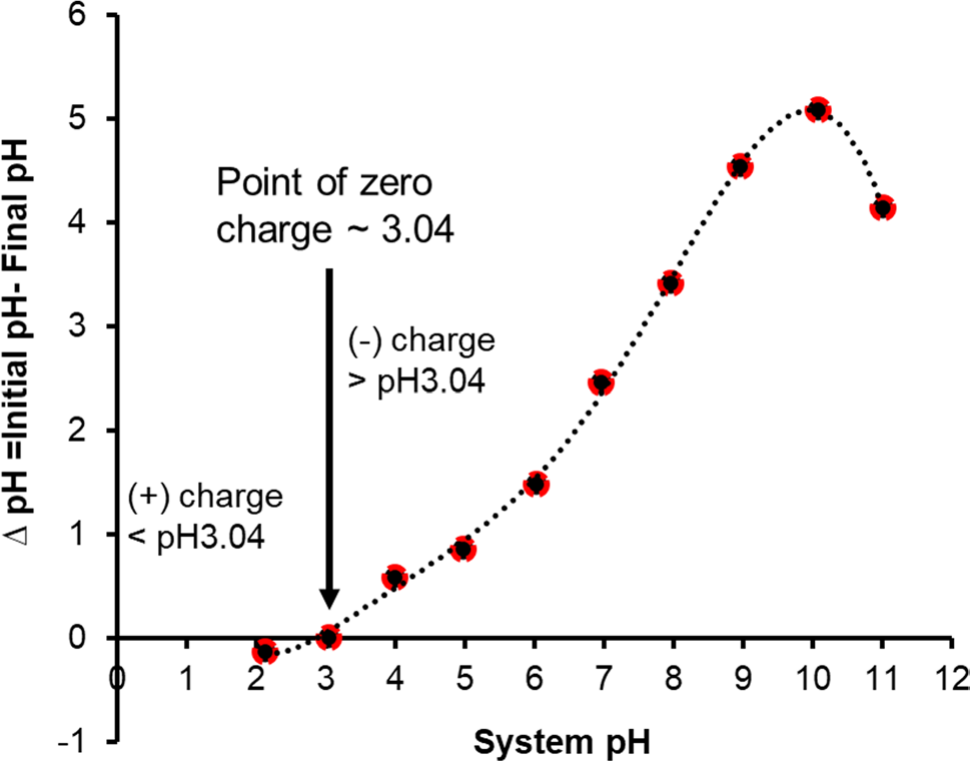
Surface charge behaviour of FeBBC

Furthermore, Fig. 9 showed that the point of zero charges (pH_pzc_) of the adsorbent is 3.04 which indicated at the pH > pH_pzc_, the adsorbents (FeBBC) are negatively charged and below the pH of pH_pzc_ FeBBC is positively charge. Whereas the pK_a_ values of IMI are 1.56 and 11.12 (Table SM1), it is reasonable that main species of imidacloprid are positive (IMI^+^) at pH below 1.56, charge neutral (IMI^±^) at pH of 3–10, and negatively charged (IMI^−^) at pH over 11.12 (Fig. SM1). Therefore, the following electrostatic interactions may occur among FeBBC and IMI.

- pH < 3: FeBCC^+^  + IMI^+^  = FeBCC^+^ ■ IMI.^+^ (electrostatic repulsion)
- pH 3–10: FeBCC^−^  + IMI^±^  = FeBCC^−^.IMI.^±^ (non-specific electrostatic interaction)
- pH > 10: FeBCC^−^  + IMI^−^  = FeBCC^−^ ■ IMI.^−^ (electrostatic repulsion)

Because of the electrostatic repulsion force, minimum sorption capacity was observed at lower pH and followed by higher pH, which is consistent with the experimental result of the solution pH (Fig. 4), FeBBC has a stable absorbed amount of IMI and increased slightly with increasing solution at pH from 3 to 10.

From the sorption kinetics, it was observed that > 50% of the total absorbed amount was absorbed onto FeBBC within 1 h (Fig. 6). Mostly, IMI is an amphiphilic molecule having a moderate hydrophobicity (logKow = 0.57), whereas the elemental compositions of FeBBC revealed its hydrophobic characteristic. Thus, it was expected that the adsorption at low pH occurred due to hydrophobic interaction among FeBBC and IMI. Figure 9 also demonstrated the amphoteric characteristics of FeBBC. Due to this behaviour, the FeBBC was able to neutralize OH^−^ which was used to set initial system pH. Hence, with increasing pH, the OH group of FeBBC also increases which facilitates H-bonding interaction among imidacloprid and biochar where the polar -NO_2_ of imidacloprid plays the vital role.

**Fig. 10:**
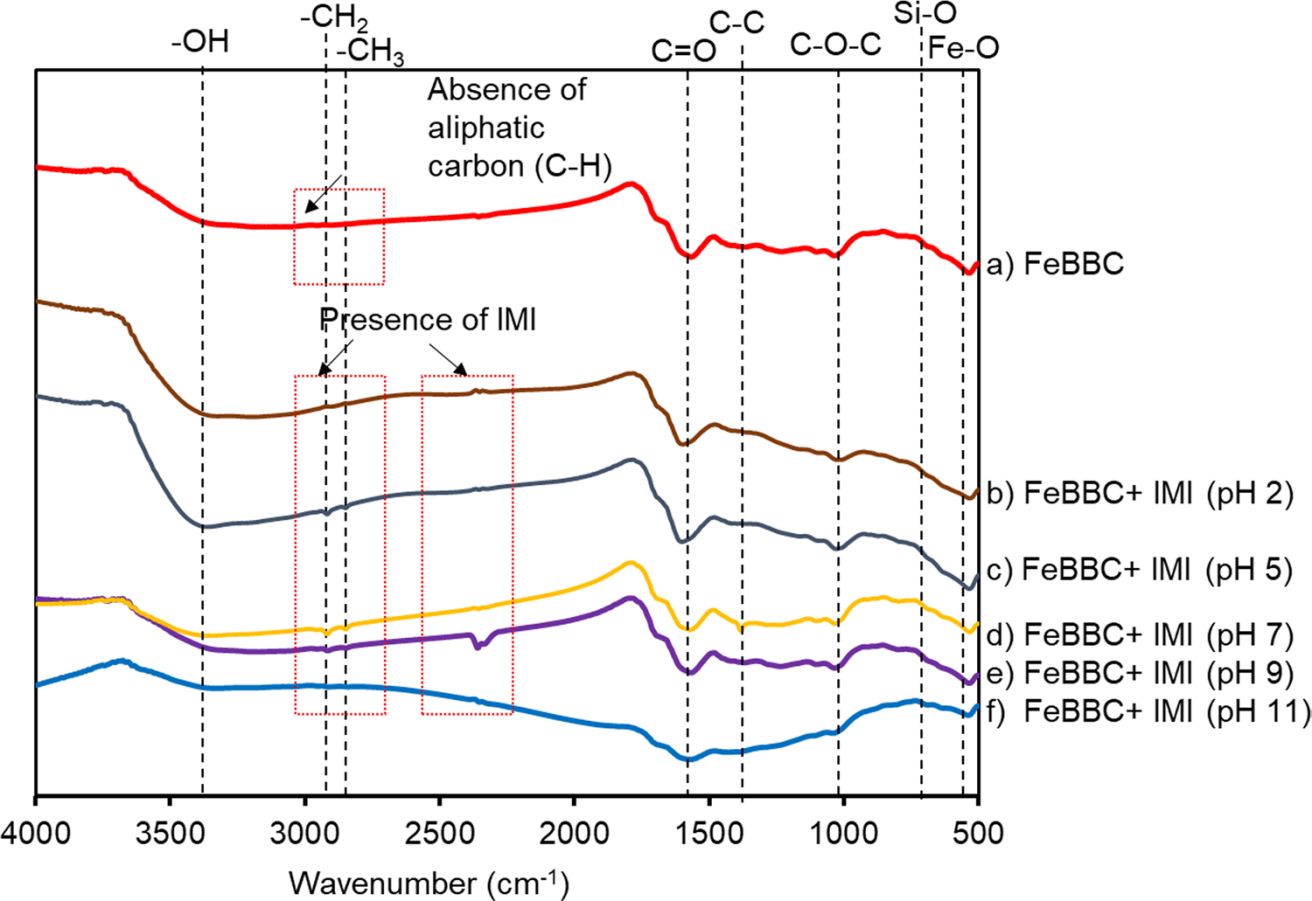
FTIR spectra of the adsorbents before and after sorption (at various initial solution pH) of IMI

The interactions among IMI and FeBBC at various pH condition was further analysed by using FTIR. The FTIR spectrum of (pre-sorption and post-sorption of IMI) are presented in Fig. 10. FeBBC has peaks at 1565, 1376, 1025.9, 674, and 528 cm^−1^ correspondence to the bending vibration of C = O, C–C, C–O–C-, Si–O, Fe–O- respectively (Luo et al. 2015) (Fig. 10). The broad peaks in 3359 cm^−1^ indicated the OH- functional groups of the carbonaceous materials. The bends at 2919 and 2888 cm^−1^ (aliphatic CH_2_ and CH_3_ bend) are absent in the FTIR spectra due to pyrolysis at high HTT (400 °C), resulting in aromatic carbon phase (Luo et al. 2014). The aromatic carbon phase is suitable for the adsorption of IMI through hydrophobic interaction. Physical sorption, including hydrophobic interaction of IMI, is a vital sorption mechanism in comparison to polar interaction for removal of IMI by FeBBC. The broad OH- stretching positioned at 3359 cm^−1^, resulting from low-affinity between O and H atom. The OH- stretching shifts leftward after IMI sorption from 3359 cm^−1^ to 3370, 3374, 3390, 3392, and 3394 cm^−1^ at solution pH 2, 5, 7, 9, and 11 respectively (Fig. 10). The pH of solution changed after adsorption as 2.50, 4.56, 4.88, 5.33, and 9.28 respectively. The OH- stretching position shift and enhancing the broadness of the peak (reduce peak sharpness) after IMI sorption demonstrated the polar interaction of IMI over the OH- functional groups of the adsorbents. New peaks were observed at 2915 cm^−1^ and 2846 cm^−1^, especially at solution pH 5, 7, at 9, demonstrated IMI sorption. Similarly, at the wavelength at 2360 and 2325 cm^−1^, a new symmetric and asymmetric bonding was observed, which resulting from IMI sorption, evidence from an earlier study, where raw FTIR spectra of IMI was found at 2328 cm^−1^ (Nuruzzaman et al. 2020).

**Fig. 11:**
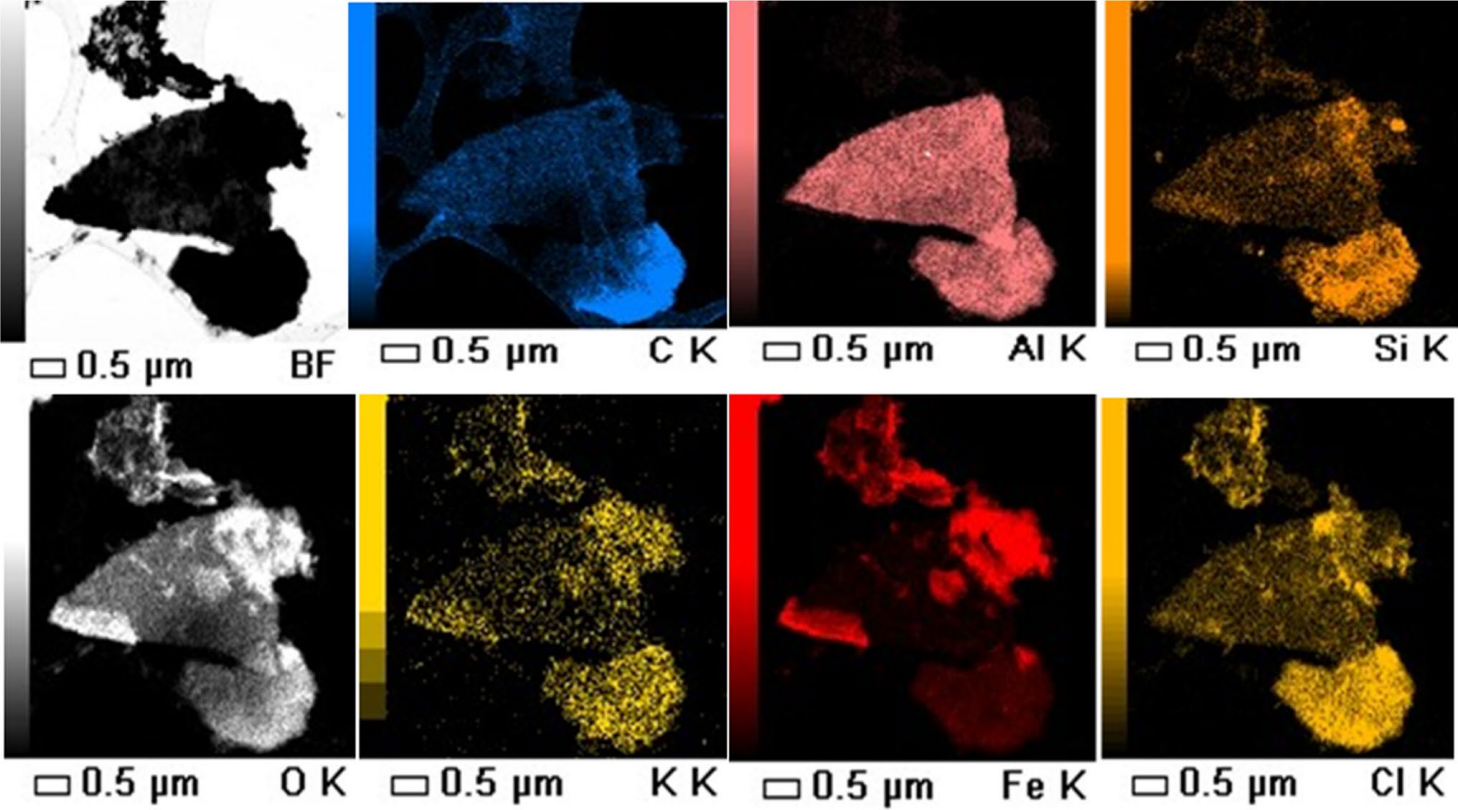
EDS elemental mapping of FeBBC after adsorption of IMI

Biochar can contain low amount of minerals’ constituents, including Al, Si, Ca, and Mg (< 5%), depending on the feedstock sources (Weber and Quicker 2018). The inorganic phase of sugarcane bagasse can provide functional groups for biochar (Si–OH, Al–OH, Si–O, Ca-O-), which could perform as active sites for charged organic molecules, including IMI (Hassan et al. 2019; Liu et al. 2013; Wang et al. 2019; Xiao et al. 2014). Elemental mapping of FeBBC (post-adsorbents) was used to visualize the elemental association and dissociation of adsorbent and adsorbate (Fig. 11). SB and BC contain carbon, oxygen, and trace amount of silicon, potassium, and aluminium. FeBBC contains additional iron apart from the existing elements in raw biochar. FeBBC (post sorption of IMI) has a substantial amount of chlorine content after adsorption of IMI. The IMI molecules contains carbon, oxygen, chlorine, and nitrogen atoms. The elemental chlorine distribution was taken place onto the FeBBC during IMI adsorption. but The chlorine had strong co-distribution with carbon, oxygen and silicon content in compare to elemental iron, and aluminum content, indicates the e dominance of ionic interaction and hydrophobic interactiontion of IMI onto the FeBBC (Fig. 11). These trace elements of biochar and iron content can perform as active sites for adsorption of IMI from water by polar interaction. As hydrophobic interaction plays a vital role in the separation of IMI from water, it has strong co-distribution with the modified biochar’s carbon and oxygen content. It has shown that iron-modified biochar performs as better adsorbents than the unmodified biochar for removing other organic and inorganic contaminants due to the induction of additional functional groups (Fe–O). The Fe–O groups are more suitable to adsorb anions than the cations under environmentally relevant pH (Beyki et al. 2016; Elgarahy et al. 2019; Li et al. 2019; Song et al. 2016; Wang et al. 2015; Yi et al. 2020; Zheng et al. 2020). Moreover, the iron or iron nanoparticles enhance lignocellulose’s catalytic degradation, enabling higher hydrophobicity, resulting in efficient removal of organic contaminants.

**Fig. 12:**
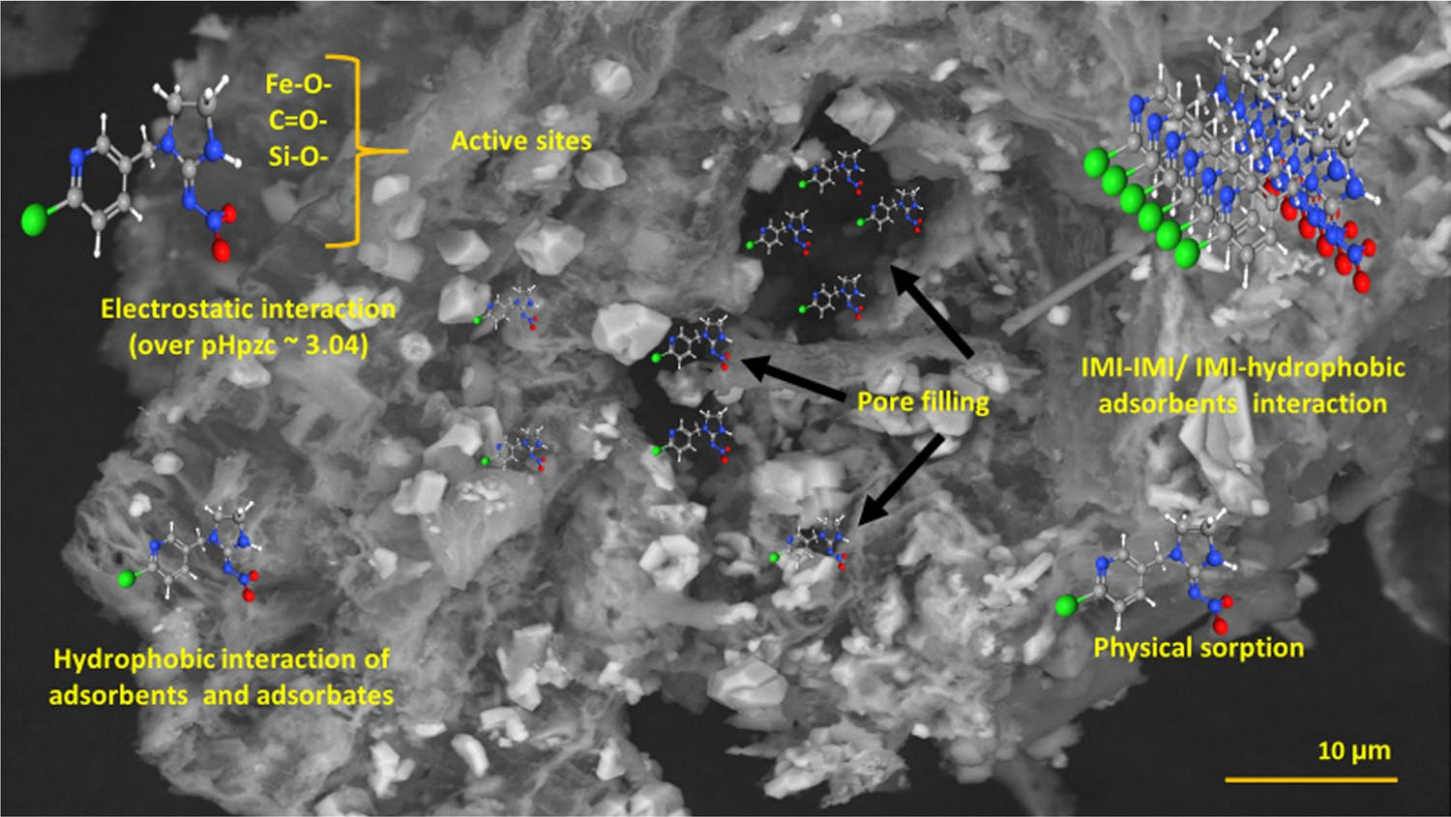
Adsorption mechanism of IMI onto iron-modified biochar (FeBBC)

Based on the isotherm, kinetics, pH study, and relevant characterization (FTIR, EDS mapping), analysis illustrated that both physical and chemical sorption mechanisms occurred to adsorb IMI from water (Fig. 12). Sorption kinetics model fitting demonstrated that physical sorption and chemisorption play a vital role in the adsorption of IMI from water. The physical sorption of IMI is highly influenced by the hydrophobicity of the materials. An insignificant effect of solution pH on adsorption of IMI demonstrated limited sorption of IMI through electrostatic interaction and ion exchange. Isotherm model indicated monolayer chemo-sorption and multilayer physical sorption of IMI from water. The oxygen-containing active sites including Fe–O groups could adsorb IMI through ionic interaction. The adsorption of IMI through liquid-film diffusion or surface diffusion is quite fast, whereas intraparticle diffusion is a time-limiting step. Probably due to this, the sorption kinetics of IMI takes a longer time to reach the equilibrium condition. The porous structure of FeBBC and its micro-porosity enable intraparticle diffusion as the primary rate-limiting step from water. IMI could form hydrogen bonding with OH-, phenolic OH-, and Fe-OH-, contained groups (Ma et al. 2021a). However, hydrogen bonding with IMI was not observed in FTIR spectra at various pH which indicated that most of the adsorption has occurred due to hydrophobic interaction.

### Comparative evaluation of IMI adsorption

Activated carbon, biochar, clay, and graphene oxide and their derivatives products are widely utilized to adsorb IMI from wastewater (Ishtiaq et al. 2020; Kalhor et al. 2018; Keshvardoostchokami et al. 2018; Ma et al. 2021c; Ma et al. 2021d; Mandal et al. 2017; Mohammad and El-Sayed 2020; Nuruzzaman et al. 2019; Singh et al. 2021). A comparative evaluation of IMI adsorption by different adsorbents is highlighted in the Table 3. The adsorption capacity largely depends on adsorbent dose, background electrolytes, and initial concentration of the adsorbates. The adsorbent dose is comparatively lower (0.05–1.0 g/L) in most studies resulting higher adsorption capacity for IMI sorption (Table 3). However, comparatively higher adsorption dose (1.67 g/L) were used in the isotherm study in this study, consequently led to less competitive adsorption capacity (10.64 mg/g) than some of the earlier report (7.78 to 330.0 mg/g). However, the removal percentage for IMI achieved as 92.67% at higher dose, which is competitive with existing reported work (Table 3).

**Table 3:** Comparative evaluation of IMI adsorption by different adsorbents

Adsorbents	Batch experiment conditions				Adsorption capacity (mg/g)	Removal %	Model fitting	IMI sorption mechanism	Reference
	C_0_ (mg/L)	t (h)	d (g/L)	pH					
Agri-waste derived biochar	1–10	24	1.0	-	–	39.9–77.8%	PFO, PSO, F, ID	Pore filling, electrostatic interaction, hydrophobic interaction	(Mandal et al. 2017)
Silver@graphene oxide nanocomposite	10	1	0.6	6.6	25.73	63%	F, PSO	Electrostatic and hydrophobic interaction	(Keshvardoostchokami et al. 2018)]
Activated carbon	10	–	–	5.2	7.78–39.37	80–99%	L, F, PFO, PSO, ID	Hydrogen bonding and π-π stacking	(Mohammad and El-Sayed 2020)
Biochar	20	6	2	–	18.17–23.79	10–65%	PFO, PSO, L, F	Ionic and hydrophobic interaction	(Zhao et al. 2018)
Biochar	20	12	0.1	–	8–15	–	L, F, PFO, PSO	Pore filling, π-π stacking, and Polar interaction	(Ma et al. 2021c)
KOH-BC	20	12	0.1	–	60–67	–	L, F, PFO, PSO	Pore filling, π-π stacking, and polar interaction	(Ma et al. 2021c)
Fe/Zn-BC	20	12	0.1	–	110–125	–	L, F, PFO, PSO	Pore filling, π-π stacking, and polar interaction	(Ma et al. 2021c)
Fe/Zn + KOH/BC	20	12	0.1	–	160–185	–	L, F, PFO, PSO	Pore filling, π-π, and polar interaction	(Ma et al. 2021c)
Magnetic biochar	20	12	0.05	–	30–330	–	L, F, PFO, PSO	Pore filling, hydrogen binding, and π-π conjugation	(Ma et al. 2021a)
Iron and base modified biochar	23.8	21	0.5–10	–	10.64	92.67%	L, F, PSO	Pore filling, polar interaction, and π-π conjugation	Present study

*Here*, *C*_*0*_, *initial concentration*; *t*, *contact time*; *d*, *adsorbent dose*; *L*, *Langmuir*; *F*, *Freundlich*; *PFO*, *pseudo-first order*; *PSO*, *pseudo-second order*; *and ID*, *intraparticle diffusion*

Biochar or modified biochar is a cost-effective adsorbent for IMI removal from wastewater. IMI sorption onto biochars is influenced by the hydrophobicity, polarity, porosity of biochar, solution pH, and contact time. Feedstock sources and pyrolysis temperature can also influence the adsorption of IMI from water. For example, biochars produced from eucalyptus bark, corn cob, bamboo chips, rice husk, and rice straw were used for IMI sorption. Of these, rice husk-derived biochar has the highest removal 39.9–77.8% of IMI from water (Mandal et al. 2017). Higher HTT is suitable for adsorption of IMI due to higher hydrophobicity of biochar generated. For example, nanoporous-activated carbons prepared at two different temperatures (300 and 500 °C) were used to remove IMI, which showed 80% and 99% removal, respectively, at pH 5.20 (Mohammad and El-Sayed 2020).

Metal-modified biochar also showed higher adsorptive performance of IMI from wastewater (Ma et al. 2021c). The sorption kinetics of IMI depends on the sorption mechanism resulting from the properties of the adsorbents. For example, liquid-film diffusion, hydrophobic interaction, and pore filling of IMI in and around the adsorbents are quite fast. On the other side, electrostatic interaction, ion exchange, and intraparticle diffusion have comparatively slow sorption kinetics. Thus, graphene oxide (hydrophobic adsorbents) has faster sorption kinetics (1 h) than the sorption kinetics of biochar or modified biochar (6–24 h) (Keshvardoostchokami et al. 2018; Ma et al. 2021c; Zhao et al. 2018) (Table 3).

In comparison to existing literature, FeBBC (prepared in this study) can remove up to ~ 92% of IMI from 23.8 mg/L (initial concentration) of IMI, where the adsorbent dose was 7.5 g/L, which is comparable. However, one major limitation of the materials is slow sorption kinetics, which requires to be improved in the future research efforts.

## Conclusion

IMI poses hazardous effects on the biotic and abiotic ecosystem that provoked to find out the solution for it’s remediation from water. Adsorption is so far established as a promising technique to remove organic contaminant. Biochar and modified biochar has been investigated as an excellent adsorbent for various contaminants. In this study, iron-modified base-activated biochar (FeBBC) were used to remove IMI from water demonstrating higher removal percentages (~ 92% at higher dosage) from the aqueous phase, which is quite remarkable as the initial concentration was relatively high (23.8 mg/L). Both physical and chemical adsorption mechanisms, including pore filling, hydrophobic interaction, and charge assisted sorption, perform efficient removal of IMI. Sorption isotherm and kinetics model fitting and effect of solution pH demonstrated both physical and chemical sorption of IMI over the adsorbents’ active sites while physical sorption performed as the primary sorption mechanism. The adsorbent is suitable for adsorption of IMI at wide pH range, which can be potentially used for IMI sorption from water. Further improvement in sorption capacity and kinetic rate will benefit the IMI removal from environmental samples. 

## Supplementary Information

Below is the link to the electronic supplementary material.

Supplementary file1 (DOCX 429 KB)

## Data Availability

All data are provided in the manuscript in the form of tables or figures.
