# Supplementary material for: Iron-modified biochar derived from sugarcane bagasse for adequate removal of aqueous imidacloprid: sorption mechanism study
Source: Environ Sci Pollut Res Int. 2022 Aug 16;30(2):4754–68. doi: 10.1007/s11356-022-22357-6 (PMC9892118; doi:10.1007/s11356-022-22357-6)
Supplement: Supplementary file 1 — Supplementary file1 (DOCX 429 KB) [file 11356_2022_22357_MOESM1_ESM.docx]

Supplementary Information (SI)

**Iron modified biochar derived from sugarcane bagasse for adequate removal of aqueous imidacloprid: sorption mechanism study**

Yongliang Chen ^a,b^, Masud Hassan ^b,c^, Md Nuruzzaman ^b,c,d^, Huiming Zhang^e^ , Ravi Naidu ^b,c^, and Yanju Liu *^b,c,d^, Ling Wang ^a^.

^a^ School of Resources and Environmental Engineering, Wuhan University of Science and Technology, Wuhan

^b^ Global Centre for Environmental Remediation, College of Engineering, Science and Environment, University of Newcastle, Callaghan, NSW 2308, Australia.

^c^ Cooperative Research Centre for Contamination Assessment and Remediation of the Environment (CRC CARE), Callaghan, NSW 2308, Australia

^d^Cooperative Research Centre for High Performance Soil (CRC SOIL), IDB Building, The University of Newcastle, Callaghan, NSW 2308, Australia

^e^Electron Microscope and X-ray (EMX) Unit, The University of Newcastle, Callaghan, NSW 2308, Australia

***Corresponding Author**

Dr. Yanju Liu, Email ID: Yanju.liu@newcastle.edu.au

Senior research fellow, Global center for environmental remediation (GCER)

Level-1, Advance Technology Center (ATC building), Ring road, University of Newcastle, NSW-2307, Australia

**Supplementary text I**

## Characterisation of the adsorbents

The elemental compositions of C, N, and S were analysed by CNS analyser (TrueMac CNS). The external morphology of the biochars was examined using a ZEISS SIGMA VP field emission scanning electron microscope (FESEM) fitted with energy-dispersive X-ray spectroscopy (EDS) system (Bruker Nano GmbH, Germany). The SEM images were captured with an accelerating voltage of 2 kV, and multiple image magnifications of various areas of the sample were obtained. Whereas the superficial element compositions were determined using EDS system with an accelerating voltage of 15 kV. Further, the distribution of Fe particles as well as carbon phase in FeBBC was investigated using a JEOL JEM-2100HR transmission electron microscope (JEOL, Japan). The TEM images were generated at 200 keV. The X-ray diffraction (XRD) pattern of the adsorbent was analysed by an X-ray diffractometer at 25 °C with cobalt (1.78 Å) anode (generated at 40 kV, 40 mA) for 3 h run time with the scan angle covered 5° < 2θ < 95°, step size 0.017° and counting time as 78.74 seconds. For understanding interfacial interaction and sorption mechanisms the adsorbent was characterised by EDS elemental colour mapping as well as Fourier-transform infrared spectroscopy (FTIR). The EDS elemental mapping was collected with 10-micron step sizes and 0.5 sec per step integration time (1.5 mm × 1.5 mm). The FTIR spectrum was recorded by using an FTIR Spectrometer (Agilent Technologies Cary 600 series) at a resolution of 8 cm^-1^ for 16 scans. The determination of the point of zero charge (pH_pzc_) of the samples was carried out following the immersion technique (Fiol &Villaescusa 2009, Kim 1973). Briefly, about 200 mg of FeBBC were added into 50 mL H_2_O (ratio of solid: liquid as 4:1) and adjusted at different pH (2-11) using 0.1 M HCl and NaOH. The suspensions were shaken in an end-over shaker for 24 h to reach equilibrium condition and pH was measured to obtain final pH. Finally, pH_pzc_ was calculated by plotting initial pH versus the change of pH (ΔpH=Initial pH-Final pH).

**Supplementary text II:**

***Isotherm Models***

Non-linear form of Langmuir isotherm model,

$$Q_{e}=\frac{q_{m}. K_{L}.C_{e}}{1+K_{L}.C_{e}}$$

Non-linear form of Freundlich isotherm model,

$$Q_{e}= K_{F}{C_{e}}^{n}$$

where, C_e_ - equilibrium solution concentration, Q_e_ – the amount of molecules adsorbed at equilibrium condition, Q_m -_ maximum adsorption capacity, K_L_ (L/mg) - constant related to the affinity between adsorbent and adsorbate, K_F_ is the Freundlich constant (K_F_ (mg/g)/(mg/L)^n^), and n (dimensionless) is a parameter that varies with the degree of heterogeneity of the adsorbing sites, reflecting the magnitude of the sorption force on surface heterogeneity.

***Kinetic models***

The linear equations of PFO model (Eq.6) (Ho &McKay 1998, Lagergren 1898), and PSO (Eq. 7) (Blanchard et al. 1984) model were represented as below:

$$ln\left( Q_{e}-Q_{t} \right)=ln\left( Q_{e} \right)-k_{1}t$$

$$1/Q_{t}=\left( \frac{1}{Qe} \right)t+\frac{1}{k_{2}Q_{e}^{2}}$$

where Q_t_ and Q_e_ (mg g^-1^) are the amounts of IMI adsorbed at any time t and equilibrium condition, respectively. k_1_ (h^−1^) and k_2_ (g/mg. h) are the pseudo–first order and pseudo–second order rate constants, respectively.

Moreover, the experimental data were also fitted with the intra-particle diffusion model (Weber &Morris 1963) to understand the diffusion mechanisms. Weber and Morris developed this model, which subsequently became known as the Weber–Morris diffusion model.

Intra-particle diffusion model:

$$Q_{t}=k_{p}t^{1/2}+C$$

where *Q_t_* (mg g^-1^) is the amount of imidacloprid adsorbed at time *t* (min), *k_p_* (mg/ g. min^1/2^) is the intra-particle diffusion rate constant and *C* is the intercept.

Scheme 1. Molecular structure of IMI (carbons, nitrogens, chlorine, oxygen and hydrogen are colored as gray, blue, green, red, and white, respectively)


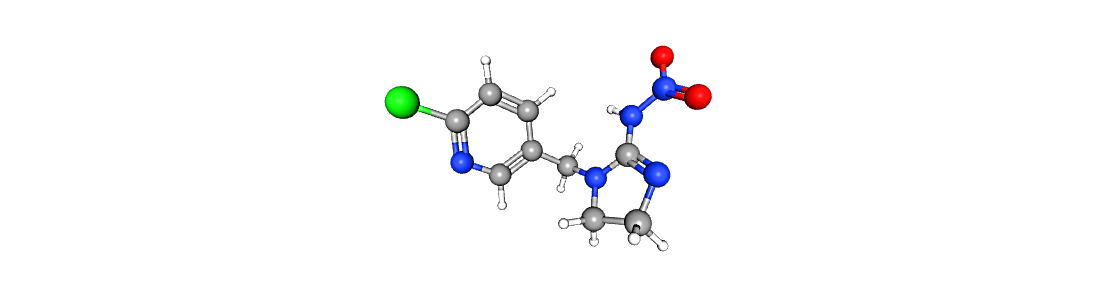


Table SM1: The physiochemical properties and molecular structure of IMI

| Parameters | Values |
| --- | --- |
| Chemical formula | [C_9_H_10_ClN_5_O_2_](https://pubchem.ncbi.nlm.nih.gov/#query=C9H10ClN5O2)) |
| Molecular wt. (g/mol) | 255.66 |
| Solubility (mg/L) | 0.61 |
| Log Kow at 21  °C | 0.57 |
| PKa_1_ | 1.56 |
| pKa_2_ | 11.12 |
| Stability to hydrolysis | pH 5 - 11 |

(National Center for Biotechnology Information. "PubChem Compound Summary for CID 86287518,Imidacloprid" PubChem, <https://pubchem.ncbi.nlm.nih.gov/compound/Imidacloprid>. Accessed 25 February, 2021)

Table SM2: Matched Phases compound, chemical formula, and minerals name of adsorbents (FeBBC)

| Ref.Code | Compound Name | Chem. Formula | Common Name | Mineral Name |
| --- | --- | --- | --- | --- |
| 98-003-4636 | Quartz low | SiO_2_ | Quartz low | Quartz low |
| 98-018-5729 | Iron | Fe1 | Iron |  |
| 98-007-6767 | Graphite 2H | C1 | Graphite 2H | Graphite 2H |


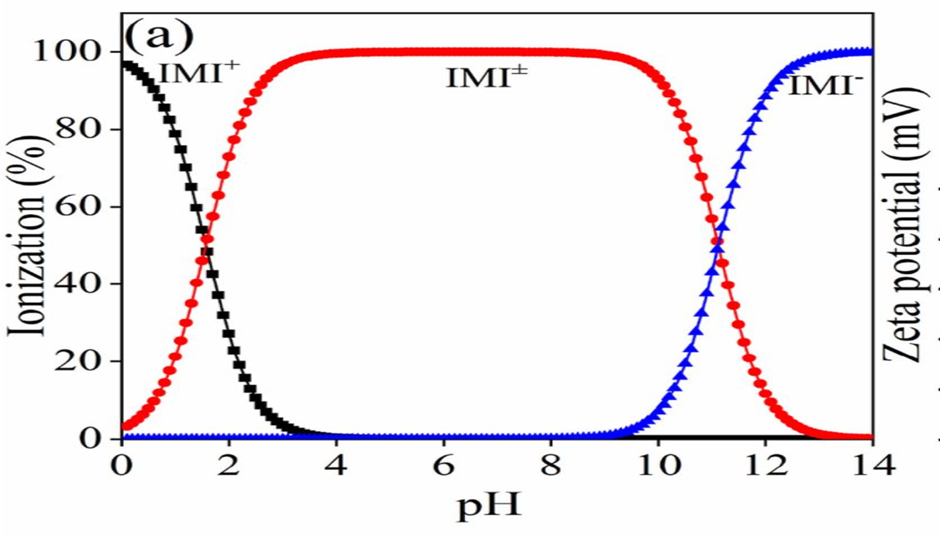


Figure SM 1: The ionization of IMI at different pH. (Reproduced along with authorization as of ref (Ma et al. 2021))

Figure SM2: Standard curve for IMI calibration


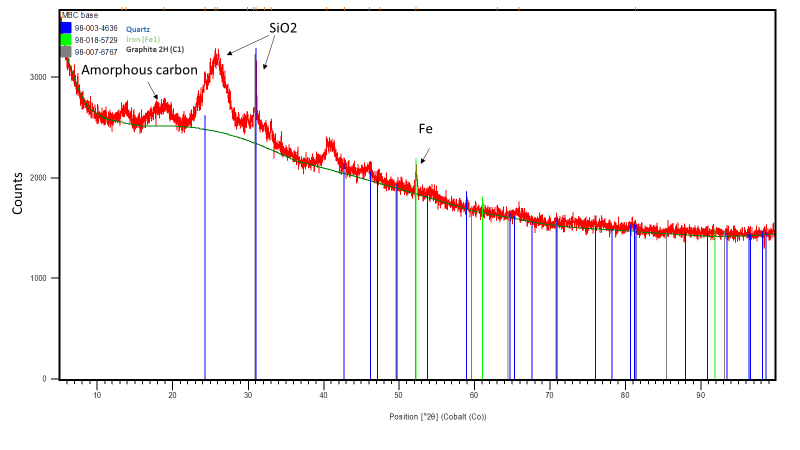


Figure SM3: XRD pattern of iron modified biochar (FeBC)

Figure SM4: The removal percentage of IMI by iron modified biochar (FeBC) at different contact time (initial IMI concentration 23.8 mg/L, dose 2.5 g/L, pH ~ 3).

**References**

Blanchard G, Maunaye M, Martin G (1984): Removal of heavy metals from waters by means of natural zeolites. Water research 18, 1501-1507

Fiol N, Villaescusa I (2009): Determination of sorbent point zero charge: usefulness in sorption studies. Environmental Chemistry Letters 7, 79-84

Ho Y-S, McKay G (1998): Sorption of dye from aqueous solution by peat. Chemical engineering journal 70, 115-124

Kim SH (1973): Immersion method for the potential of zero charge determination. Electrode pretreatment. The Journal of Physical Chemistry 77, 2787-2789

Lagergren SK (1898): About the theory of so-called adsorption of soluble substances. Sven. Vetenskapsakad. Handingarl 24, 1-39

Ma Y, Qi Y, Yang L, Wu L, Li P, Gao F, Qi X, Zhang Z (2021): Adsorptive removal of imidacloprid by potassium hydroxide activated magnetic sugarcane bagasse biochar: Adsorption efficiency, mechanism and regeneration. Journal of Cleaner Production 292, 126005

Weber WJ, Morris JC (1963): Kinetics of adsorption on carbon from solution. Journal of the Sanitary Engineering Division 89, 31-60
